# Dual control of MAPK activities by AP2C1 and MKP1 MAPK phosphatases regulates defence responses in Arabidopsis

**DOI:** 10.1093/jxb/erac018

**Published:** 2022-01-28

**Authors:** Zahra Ayatollahi, Vaiva Kazanaviciute, Volodymyr Shubchynskyy, Kotryna Kvederaviciute, Manfred Schwanninger, Wilfried Rozhon, Michael Stumpe, Felix Mauch, Sebastian Bartels, Roman Ulm, Salma Balazadeh, Bernd Mueller-Roeber, Irute Meskiene, Alois Schweighofer

**Affiliations:** 1 Max Perutz Labs, University of Vienna, Vienna BioCenter, Dr.-Bohr-Gasse 9, A-1030 Vienna, Austria; 3 Institute of Biotechnology, Life Sciences Center, Vilnius University, Sauletekio al. 7, LT-10257 Vilnius, Lithuania; 4 Department of Chemistry, University of Natural Resources and Applied Life Sciences, Muthgasse 18, A-1190 Vienna, Austria; 5 Department of Agriculture, Ecotrophology, and Landscape Development, Anhalt University of Applied Sciences, Strenzfelder Allee 28, D-06406 Bernburg, Germany; 6 Department of Biology, University of Fribourg, Chemin du Musée 10, CH-1700 Fribourg, Switzerland; 7 Faculty of Biology, Institute of Biology II, University of Freiburg, Schänzlestraße 1, D-79104 Freiburg, Germany; 8 Department of Botany and Plant Biology, Section of Biology, Faculty of Sciences, University of Geneva, 30 Quai E. Ansermet, CH-1211 Geneva, Switzerland; 9 Max-Planck-Institute of Molecular Plant Physiology (MPIMP), Am Mühlenberg 1, D-14476 Potsdam, Germany; 10 University of Potsdam, Karl-Liebknecht-Straße 24, D-14476 Potsdam, Germany; 11 Institute of Biology Leiden (IBL), Leiden University, Sylviusweg 72, 2333 BE Leiden, The Netherlands; 12 Center of Plant Systems Biology and Biotechnology (CPSBB), Ruski 139 Blvd., Plovdiv 4000, Bulgaria; 13 Department of Functional and Evolutionary Ecology, University of Vienna, Djerassiplatz 1, A-1030 Vienna, Austria; 14 Queen’s University, Canada

**Keywords:** ANAC, AP2C1, Arabidopsis, dual-specificity phosphatase, mitogen-activated protein kinase, MKP1, Ser/Thr protein phosphatase of the type 2C, WRKY

## Abstract

Mitogen-activated protein kinase (MAPK) cascades transmit environmental signals and induce stress and defence responses in plants. These signalling cascades are negatively controlled by specific Ser/Thr protein phosphatases of the type 2C (PP2C) and dual-specificity phosphatase (DSP) families that inactivate stress-induced MAPKs; however, the interplay between phosphatases of these different types has remained unknown. This work reveals that different Arabidopsis MAPK phosphatases, the PP2C-type AP2C1 and the DSP-type MKP1, exhibit both specific and overlapping functions in plant stress responses. Each single mutant, *ap2c1* and *mkp1*, and the *ap2c1 mkp1* double mutant displayed enhanced stress-induced activation of the MAPKs MPK3, MPK4, and MPK6, as well as induction of a set of transcription factors. Moreover, *ap2c1 mkp1* double mutants showed an autoimmune-like response, associated with increased levels of the stress hormones salicylic acid and ethylene, and of the phytoalexin camalexin. This phenotype was reduced in the *ap2c1 mkp1 mpk3* and *ap2c1 mkp1 mpk6* triple mutants, suggesting that the autoimmune-like response is due to MAPK misregulation. We conclude that the evolutionarily distant MAPK phosphatases AP2C1 and MKP1 contribute crucially to the tight control of MAPK activities, ensuring appropriately balanced stress signalling and suppression of autoimmune-like responses during plant growth and development.

## Teaser

Mitogen-activated protein kinase (MAPK) phosphatase double mutant *ap2c1 mkp1* Arabidopsis plants exhibit constitutive, autoimmune-like stress responses, dependent on their substrate MAPKs MPK3 and MPK6.

## Introduction

Reversible protein phosphorylation is one of the most commonly used mechanisms for the molecular transmission of stress signals and developmental cues. This mechanism is based on the opposing actions of protein kinases and protein phosphatases. Mitogen-activated protein kinases (MAPKs) are highly conserved major components of developmental and stress signalling cascades in eukaryotes. MAPKs are activated by upstream MAPK kinases via phosphorylation of Thr and Tyr residues within their activation loop. This activation eventually leads to the reprogramming of cellular activities, including the modulation of gene expression, to generate appropriate responses. The activation of MAPKs does not represent a simple on/off switch, as both the magnitude and duration of activation are crucial for determining the signalling outcome (Marshall, 1995). Prolonged or constant activation of a MAPK cascade can have detrimental effects, as illustrated by the hypersensitive response-induced cell death in plants expressing a constitutively active version of MAPK kinase (Ren *et al.*, 2002; Liu *et al.*, 2007). Thus, negative regulation and inactivation mechanisms are important for the correct cellular response. Specific protein phosphatases can dephosphorylate and thereby inactivate MAPKs. As dual phosphorylation of the Thr-X-Tyr motif in the activation loop is required for MAPK activation (Caunt and Keyse, 2013), dephosphorylation of either phospho-amino acid residue inactivates the MAPK and inhibits downstream signalling. Interestingly, this inactivation can be accomplished by evolutionarily distant protein phosphatases, including Ser/Thr protein phosphatases of the type 2C (PP2C) MAPK phosphatases (Schweighofer *et al.*, 2004; Schweighofer *et al.*, 2007; Fuchs *et al.*, 2013) and PTP-type dual specificity (Tyr and Ser/Thr) phosphatases (DSPs) (Bartels *et al.*, 2010; Jiang *et al.*, 2018). However, their interplay is presently unknown.

*Arabidopsis thaliana* DSP-type MAPK phosphatase 1 (MKP1) interacts with the stress-responsive MAPKs MPK3, MPK4, and MPK6, and controls their activities (Ulm *et al.*, 2002; Bartels *et al.*, 2009; Anderson *et al.*, 2011). The *mkp1* knockout mutant is hypersensitive to genotoxic stress, including UV-B radiation (Gonzalez Besteiro and Ulm, 2013*a*, *b*), but is more resistant than the wild type (WT) to the virulent bacterial pathogen *Pseudomonas syringae* pv. *tomato* (*Pto*) (Bartels *et al.*, 2009; Anderson *et al.*, 2011; Anderson *et al.*, 2014; Escudero *et al.*, 2019). Specifically in Arabidopsis Columbia accession, *mkp1* shows an autoimmune-like growth phenotype dependent on the disease resistance gene homologue *SUPPRESSOR OF npr1-1, CONSTITUTIVE 1* (*SNC1*), and is associated with enhanced MAPK activities (Bartels *et al.*, 2009). The phospho-Tyr-specific PTP-type protein phosphatase PTP1 also interacts with MPK6 and MPK3 in transient assays. The *mkp1 ptp1* double mutant exhibits up-regulation of MPK6-dependent plant defence responses and a further enhanced autoimmune-like phenotype (Bartels *et al.*, 2009).

A group of PP2C-type phosphatases, including AP2C1, interacts with MAPKs and controls their activities (Schweighofer *et al.*, 2007; Umbrasaite *et al.*, 2010). *AP2C1* is induced by wounding and biotic stress, and functions as a negative regulator of MPK3, MPK4, and MPK6, controlling levels of wound-induced jasmonate and ethylene (ET) as well as plant immunity (Schweighofer *et al.*, 2007; Galletti *et al.*, 2011; Sidonskaya *et al.*, 2016; Shubchynskyy *et al.*, 2017). *ap2c1* plants do not display obvious developmental phenotypes under standard growth conditions (Schweighofer *et al.*, 2007), implying a specific function of AP2C1 under stress conditions and the contribution of other, presently unknown, MAPK phosphatases for MAPK control in the absence of AP2C1.

Activation of transcription factors (TFs) and changes in gene expression are part of the cellular response to a perceived signal in order to reprogram cellular processes (Rauf *et al.*, 2013). A number of TFs, including WRKY and AP2-domain/ethylene-responsive factor (AP2/ERF) family members, have been suggested or demonstrated to act downstream of MAPKs in plants (Asai *et al.*, 2002; Kim and Zhang, 2004; Menke *et al.*, 2005; Nakano *et al.*, 2006; Popescu *et al.*, 2009; Bethke *et al.*, 2009; Mao *et al.*, 2011; Li *et al.*, 2012; Meng and Zhang, 2013; Guan *et al.*, 2014). Subsequently, these proteins may constitute an important link between pathogen- or wound-induced MAPK signalling and downstream transcriptional reprogramming.

Considering the broad spectrum of signals transmitted by the same MAPKs (Rodriguez *et al.*, 2010; Meng and Zhang, 2013), such as MPK6, it is puzzling how the specificity of the responses for perceived stimuli is generated. The phylogenetic diversity and distinct enzymatic mechanisms of protein phosphatases that are able to inactivate MAPKs support the idea of a contribution of MAPK phosphatases to the versatility and specificity of MAPK networks. Here, we investigate the roles of the phylogenetically distant Arabidopsis MAPK phosphatases AP2C1 and MKP1 and, in particular, their functional redundancies. We show that AP2C1 and MKP1 together repress plant autoimmune-like responses, including salicylic acid (SA) and ET accumulation, and early senescence. These observations in the *ap2c1 mkp1* double mutant are underlined by the mis-expression of specific TF genes, including members of the *WRKY*, *AP2/ERF*, and Arabidopsis NAM, ATAF, and CUC (*ANAC*) families whose expression is at least partially mediated by MPK6 and MPK3.

## Materials and methods

### Plant lines, genetic crosses, and growth conditions

All plant lines used in this study were in the *Arabidopsis thaliana* accession Columbia (Col-0), with *mkp1* being an introgression line from a Wassilewskija background (Bartels *et al.*, 2009). The T-DNA insertion line *ap2c1* (SALK_065126) (Schweighofer *et al.*, 2007) was crossed with the T-DNA insertion lines *ptp1* (SALK_118658) and *mkp1* to generate the *ap2c1 ptp1* and *ap2c1 mkp1* double mutants, respectively*. mpk6-2* (SALK_073907) and *mpk3-1* (SALK_151594) were used for genetic crosses generating *ap2c1 mkp1 mpk6* and *ap2c1 mkp1 mpk3* triple mutants. The T-DNA insertion lines *ap2c2* (GABI-Kat_316F11) and *ap2c3* (SALK_109986) (Umbrasaite *et al.*, 2010) were crossed with *mkp1* to generate *ap2c2 mkp1* and *ap2c3 mkp1* double mutants. Combinatorial mutants were identified in the F_2_ generation and also confirmed in subsequent generations by PCR genotyping using T-DNA- and gene-specific primers (Schweighofer *et al.*, 2007; Bartels *et al.*, 2009; Umbrasaite *et al.*, 2010; Shubchynskyy *et al.*, 2017). For protein and RNA extraction, and for measurements of ET and SA, plants were grown on soil for 5–7 weeks in a phytotron chamber under short-day conditions (8 h light, 22 °C/16 h dark, 20 °C).

For experiments at the seedling stage, seeds were surface sterilized and spread on plates containing half-strength Murashige and Skoog (MS) medium (Duchefa), pH 5.7, 1% (w/v) sucrose, and 0.7% plant agar (w/v; Duchefa). Seedlings were grown under long-day conditions (16 h light/8 h dark) at 22 °C. For suppression of growth phenotypes, plants were kept under short-day conditions in a tray with closed lid at 24 °C with saturating relative humidity.

### *Ex vivo* kinase activity assay and MAPK immunoblotting

Plant protein extraction and the *ex vivo* kinase assay were performed as described previously (Schweighofer *et al.*, 2007; Schweighofer *et al.*, 2009) using polyclonal antibodies for immunoprecipitation and myelin basic protein (MBP) as the *in vitro* substrate of immunoprecipitated MAPKs. A master mix containing MBP and γ-ATP was used for each series of kinase assays to ensure equal distribution of the substrate to all reactions. Treatment with the pathogen-associated molecular pattern (PAMP) flg22 was performed as described previously (Shubchynskyy *et al.*, 2017). Signal intensities of phosphorylated MBP were quantified with ImageQuant software (version 5.1; Amersham). MAPK protein amounts were visualized with the antibodies Anti-AtMPK3 (M8318), Anti-MPK4 (A6979), and Anti-AtMPK6 (A7104) (all Sigma).

### RNA extraction and quantitative reverse transcription–PCR

Total RNA from leaves was isolated with the RNeasy Plant Mini Kit (Qiagen) and treated with TURBO DNA-free DNase I (Ambion) according to the manufacturers’ instructions. RNA integrity was checked on 1% (w/v) agarose gels and the concentration was measured before and after DNAse I digestion. The absence of genomic DNA was verified by PCR using primers targeting an intron of the control gene *At5g65080*. cDNA synthesis was performed using the First Strand cDNA Synthesis Kit (Thermo Scientific). The efficiency of cDNA synthesis was estimated by quantitative reverse transcription–PCR (RT–qPCR) analysis using a primer pair amplifying the 3ʹ part of the control gene encoding GAPDH and a primer pair amplifying the 5ʹ part of the same gene. RT–qPCR reactions were performed as described previously (Balazadeh *et al.*, 2008). *ACTIN2* was selected as a reference gene for which four replicates were measured in each PCR run, and their average cycle threshold (CT) was used for relative expression analyses. TF expression data were normalized by subtracting the mean *ACTIN2* gene CT value from the CT value (ΔCT) of each gene of interest. The expression value in the comparison between different genotypes was calculated using the expression 2^–ΔΔCT^, where ΔΔCT represents the ΔCT of the mutant of interest minus the ΔCT of the control (WT). For TF expression profiling, an advanced version of an expression profiling platform (Balazadeh *et al.*, 2008) that was originally described by Czechowski *et al.* (2004) was used, covering 1880 Arabidopsis TF genes. Statistical analysis was performed with JASP software (https://jasp-stats.org; version 0.14.1).

### ET measurements and quantification of total SA and camalexin

Measurements of ET were performed by gas chromatography (Hewlett Packard 5890 Series II) with an Al_2_O_3_ column (Agilent Technologies). Whole rosettes of 4-week-old plants grown under long-day conditions were collected, and leaves were wounded and transferred into 20 ml vials containing 4 ml half-strength MS medium with 0.8% (w/v) plant agar, to reduce the volume of the head space, and the vials were sealed so they were airtight. After 24 h, 100 µl of the gas phase was taken from the vials and analysed by gas chromatography–flame ionization detection. ET production was calculated per milligram of fresh tissue per hour.

Total SA was quantified as described previously (Rozhon *et al.*, 2005) except that 20 µM EDTA was added to the HPLC eluent. Camalexin levels were determined as described previously (Shubchynskyy *et al.*, 2017).

## Results

### *ap2c1 mkp1* double mutant plants show growth and developmental defects that are at least partially mediated by MPK6 and MPK3

**Fig. 1.**
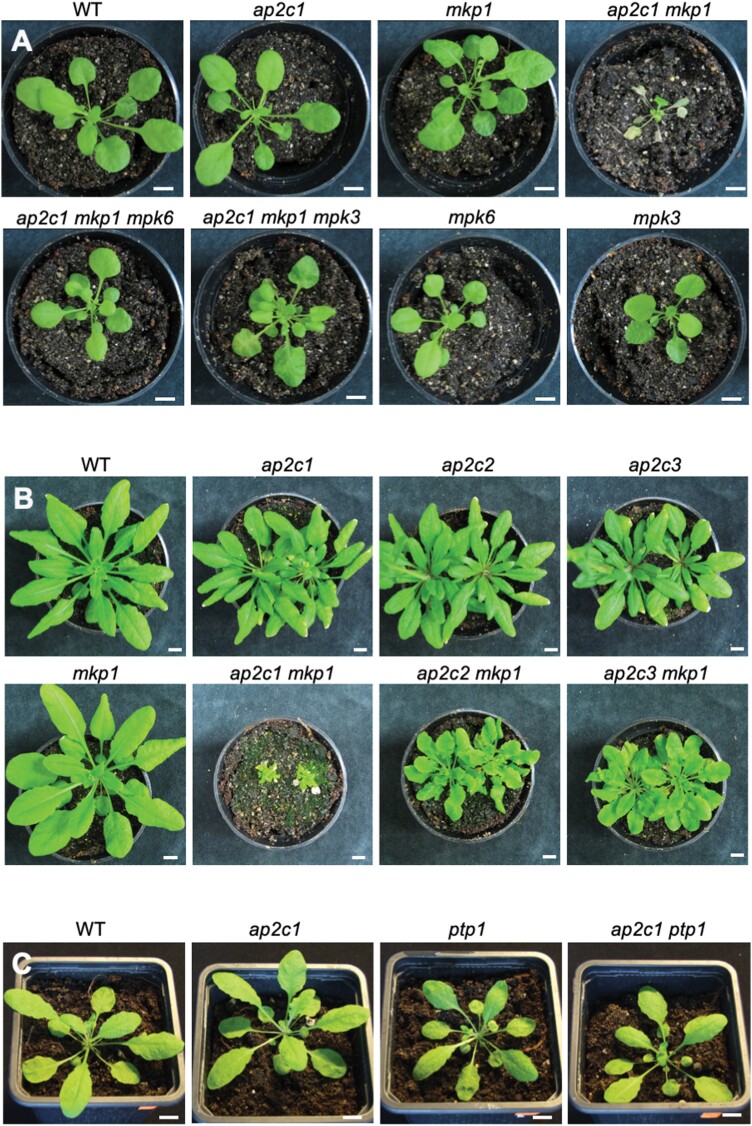
Loss of both AP2C1 and MKP1 causes developmental defects and precocious cell death, mediated by MPK6 and MPK3. (A) Phenotypes of 5-week-old plants of the indicated genotypes grown under standard short-day conditions. (B) Phenotypes of plants of the indicated genotypes grown for 4 weeks under short-day conditions followed by 3 weeks under long-day conditions. (C) Phenotypes of plants of the indicated genotypes grown for 6 weeks under short-day conditions. Scale bars=1 cm.

To investigate the specific and/or overlapping roles of the MAPK phosphatases AP2C1 and MKP1, we took advantage of the Arabidopsis T-DNA insertion knockout mutants *ap2c1* and *mkp1*, respectively (Schweighofer *et al.*, 2007; Bartels *et al.*, 2009). Phenotypically, *ap2c1* and *mkp1* mutant plants did not show any difference from WT plants when grown for up to 5 weeks under short-day conditions (Fig. 1A). However, *mkp1* plants grown under long-day conditions showed altered morphology, such as aberrant leaf development and early senescence, which appeared approximately 3 weeks after germination (Supplementary Fig. S1A), as described previously (Bartels *et al.*, 2009).

To further analyse the functions of AP2C1 and MKP1 in plants, we generated a double mutant by genetic crossing. *ap2c1 mkp1* plants showed phenotypic differences from both the WT and the single mutants. These appeared about 2 weeks after germination under standard growth conditions in soil in all *ap2c1 mkp1* double mutants; first their sizes started differing, and during further growth *ap2c1 mkp1* plants developed more pronounced multiple defects, including severe dwarfism and aberrant leaf development (Fig. 1A, B; Supplementary Fig. S1B–G). Four weeks after germination, phenotypic abnormalities became even more evident, including early senescence, spontaneous macroscopic lesions, and abnormal leaf morphology. These developmental defects were suppressed when plants were grown under conditions of elevated humidity and increased temperature (Supplementary Fig. S2), indicating a dependency on environmental cues. However, during flowering, misshapen inflorescences and strongly reduced fertility were always observed (Supplementary Fig. S1E). These phenotypes were specific for *ap2c1 mkp1* plants, as crossing *ap2c1* with *ptp1* did not lead to phenotypic alterations (Fig. 1C) compared with *mkp1 ptp1* (Bartels *et al.*, 2009). Crossing *mkp1* with either of two other clade B AP2C mutants, *ap2c2* or *ap2c3* (Umbrasaite *et al.*, 2010), led to only mild phenotypes compared with the strong defects of *ap2c1 mkp1* plants (Fig. 1B).

Since MPK6 and MPK3 are targets of AP2C1 and MKP1 (Ulm *et al.*, 2002; Schweighofer *et al.*, 2007; Galletti *et al.*, 2011), we addressed the impact of MPK6 and MPK3 on the phenotypic aberrations detected in *ap2c1 mkp1* plants. To this end, *ap2c1 mkp1 mpk6* and *ap2c1 mkp1 mpk3* triple mutant plants were created and their phenotypes were compared with those of *ap2c1 mkp1*. The phenotype of *ap2c1 mkp1 mpk6* plants was more similar to that of the WT than that of *ap2c1 mkp1*. The loss of MPK6 suppressed most phenotypic defects observed in *ap2c1 mkp1*, such as extreme dwarfism, aberrant leaf shapes, premature leaf senescence, and impaired fertility (Fig. 1A, Supplementary Fig. S3). However, at later developmental stages, *ap2c1 mkp1 mpk6* plants still appeared overall smaller than WT and displayed senescence in the older leaves (Supplementary Fig. S3E, M). Similarly, *ap2c1 mkp1 mpk3* plants suppressed the observed *ap2c1 mkp1* phenotypes in early development (Fig. 1A) but to a lesser extent at later developmental stages compared with *ap2c1 mkp1 mpk6* (Supplementary Fig. S3F, N). Both *ap2c1 mkp1 mpk6* and *ap2c1 mkp1 mpk3* developmental phenotypes were suppressed during growth in conditions of elevated temperature and increased humidity, as observed with *ap2c1 mkp1* (Supplementary Fig. S2).

Overall, these results suggest that AP2C1 and MKP1 protein phosphatases act partially redundantly and that the presence of at least either gene is necessary for normal plant development. The phenotypes observed in *ap2c1 mkp1* plants are predominantly MPK6- and MPK3-dependent.

### Dual control of stress-induced MAPK activities by AP2C1 and MKP1

**Fig. 2.**
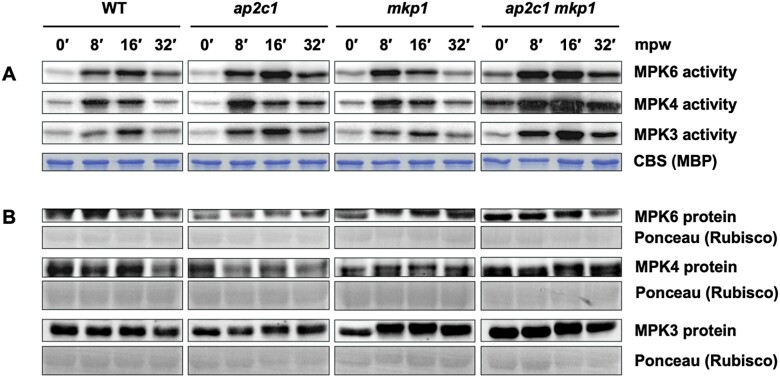
AP2C1 and MKP1 control wound-induced MAPK activities. Analysis of wound-induced MPK6, MPK4, and MPK3 kinase activities and protein amounts of leaves from 6-week-old WT, *ap2c1*, *mkp1*, and *ap2c1 mkp1* plants grown under short-day conditions. (A) MAPK activities were determined after immunoprecipitation by phosphorylation of MBP detected by autoradiography. The entire kinase assay was based on one common master mix containing MBP and γ-ATP. Loading is demonstrated by Coomassie blue staining (CBS); representative lanes are shown. The experiment was repeated twice with similar results. (B) MAPK protein amounts before and after wounding demonstrated by immunoblotting of MPK3, MPK4, and MPK6 from total protein extract using specific antibodies. Loading is demonstrated by Ponceau S staining (Rubisco protein). mpw, Minutes post wounding.

Our previous work revealed the involvement of AP2C1 in the regulation of MAPK activities induced by wounding, nematode feeding, and PAMPs (Schweighofer *et al.*, 2007; Sidonskaya *et al.*, 2016; Shubchynskyy *et al.*, 2017). To check for a potential overlapping role of MKP1, we first analysed MPK3, MPK4, and MPK6 activities after wounding the leaves of WT plants and of the single mutant plants *ap2c1* and *mkp1*. Kinase activities were assayed after immunoprecipitation from total protein extracts using specific antibodies. In agreement with our previous findings (Schweighofer *et al.*, 2007), *ap2c1* plants showed higher and more sustained wound-induced activities of MPK3, MPK4, and MPK6 compared with the WT (Fig. 2). Interestingly, MPK4 activity was more intense and sustained in *ap2c1* compared with *mkp1* plants, indicating a specific role of AP2C1 in the regulation of MPK4 during wounding. MPK3 activity in *ap2c1* plants was slightly higher and more sustained in comparison to WT and *mkp1*. In *mkp1* plants, the peak activity of MPK6 was shifted to an earlier time point compared with the WT. In *ap2c1 mkp1* plants, we detected strongly and moderately enhanced basal activity of MPK4 and MPK6, respectively, whereas the basal activity of MPK3 was not affected in comparison to the WT or single mutant lines. The stronger and more sustained wound-induced activation of MAPKs observed in single-mutant plants was additionally enhanced in the double mutant *ap2c1 mkp1* (Fig. 2, Supplementary Fig. S4A). The MPK3, MPK4, and MPK6 protein amounts were comparable in both the single and double mutants and the WT (Fig. 2). Treatment of WT, *ap2c1* and *mkp1* single and double mutants, and *ap2c1 mkp1 mpk3* and *ap2c1 mkp1 mpk6* triple mutant plants with the PAMP flg22 led to similar MAPK activation patterns as those observed after wounding, with increased and prolonged MAPK activities in the *ap2c1 mkp1* plants compared with the single mutants and the WT (Supplementary Fig. S4B, C).

*MKP1* has been reported to be constitutively expressed (Ulm *et al.*, 2002), whereas *AP2C1* is transcriptionally responsive to stress (Schweighofer *et al.*, 2007; Sidonskaya *et al.*, 2016). We tested whether reciprocal compensational expression may occur under long-day conditions and thus analysed *AP2C1* and *MKP1* mRNA levels in the *mkp1* and *ap2c1* mutants, respectively. RT–qPCR analyses showed only very slightly increased expression of *MKP1* in *ap2c1* plants, whereas the expression of *AP2C1* was approximately 160% of the WT level in the *mkp1* plants (Supplementary Fig. S5), suggesting a compensatory transcriptional activation of *AP2C1* in the absence of MKP1. Our results suggest cooperative action and partial redundancy in the regulation of MAPKs by these two evolutionarily distant and unrelated MAPK phosphatases.

### AP2C1 and MKP1 play partially redundant roles in the control of wound-induced ET synthesis

**Fig. 3.**
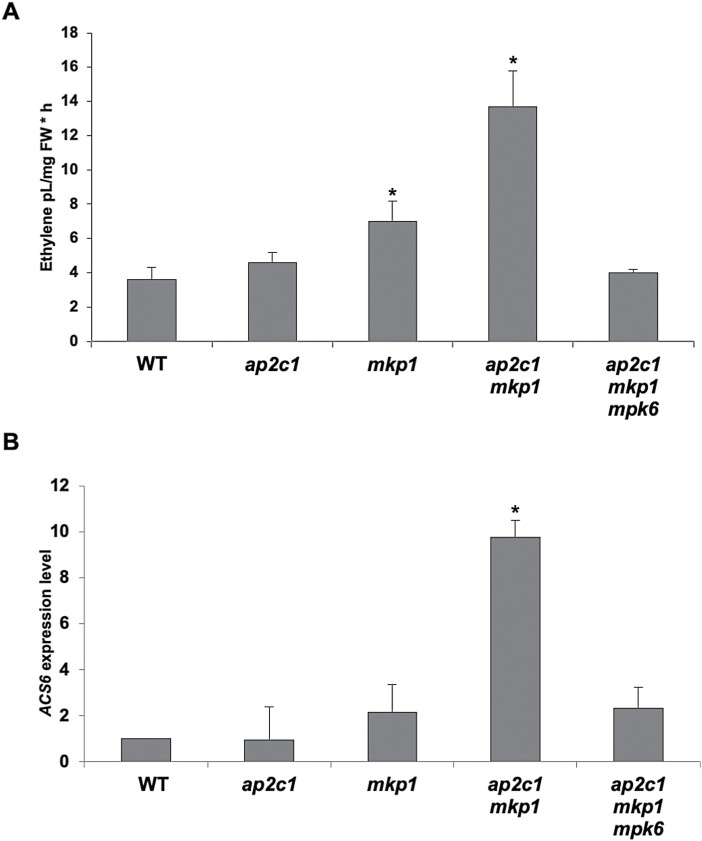
*ap2c1 mkp1* plants have higher *ACS6* expression and produce more ET upon wounding than WT plants, mainly mediated by MPK6. (A) ET levels produced by 4-week-old plants of the indicated genotypes grown under standard long-day conditions. ET amounts are shown as pl mg^–1^ plant fresh weight (FW) per hour. Bars represent mean ±SD values of three biological replicates. ∗*P*<0.05 (Student’s *t*-test). (B) RT–qPCR analysis of *ACS6* expression in leaves of 6-week-old plants of the indicated genotypes grown under short-day conditions; expression levels in the WT are set to 1. Bars represent mean ±SD values of three biological replicates. ∗*P*<0.05 (Student’s *t*-test).

Enhanced ET production is an early response of plants subjected to biotic or abiotic stresses (Wang *et al.*, 2002; Ju and Chang, 2012). We have previously shown that ectopic expression of *AP2C1* suppresses MPK6 activation and wound-induced ET production in plant leaves (Schweighofer *et al.*, 2007). Since both AP2C1 and MKP1 control MPK6 activity, a major determinant in the regulation of ET biosynthesis (Liu and Zhang, 2004; Li *et al.*, 2012), we analysed the amounts of wound-induced ET in leaves of WT, *ap2c1*, *mkp1*, *ap2c1 mkp1*, and *ap2c1 mkp1 mpk6* plants. As reported previously (Schweighofer *et al.*, 2007), the wound-induced ET amounts were similar in *ap2c1* and WT plants (Fig. 3A). However, significantly greater (*P*<0.05) amounts of ET accumulated in wounded *mkp1* plants and even higher amounts in the *ap2c1 mkp1* double mutant (Fig. 3A). Our data suggest a primary role of MKP1 in the control of wound-triggered ET production and that although disruption of AP2C1 alone is not sufficient to alter ET production upon wounding, it contributes significantly to the regulation of ET amounts in the absence of MKP1. Interestingly, and in agreement with the overall milder phenotype, wound-induced ET accumulation in *ap2c1 mkp1 mpk6* plants was similar that detected in the WT (Fig. 3A).

The transcriptional regulation of 1-aminocyclopropane-1-carboxylic synthase (ACS) enzymes contributes to the control of ET production (Li *et al.*, 2012). Therefore, we quantified the transcripts of *ACS6,* the expression of which is significantly induced after pathogen attack (Li *et al.*, 2012) and wounding (Li *et al.*, 2018). Compared with the WT, no changes in *ACS6* transcript levels were detected in *ap2c1*, slightly higher levels in *mkp1*, and a 9-fold increase in *ap2c1 mkp1*, whereas in *ap2c1 mkp1 mpk6* plants levels were not significantly different from those in WT plants (Fig. 3B). Thus, our data show that *ACS6* is more strongly expressed in *ap2c1 mkp1* plants, which likely contributes to the increased amounts of ET upon wounding, and that both effects are mediated by MPK6. Taken together, the wound-induced MAPK activities, expression patterns, and effects on ET production suggest that AP2C1 and MKP1 have both distinct and overlapping functions in wounded leaves.

### Transcription factor gene expression is deregulated in *ap2c1 mkp1* plants

**Fig. 4.**
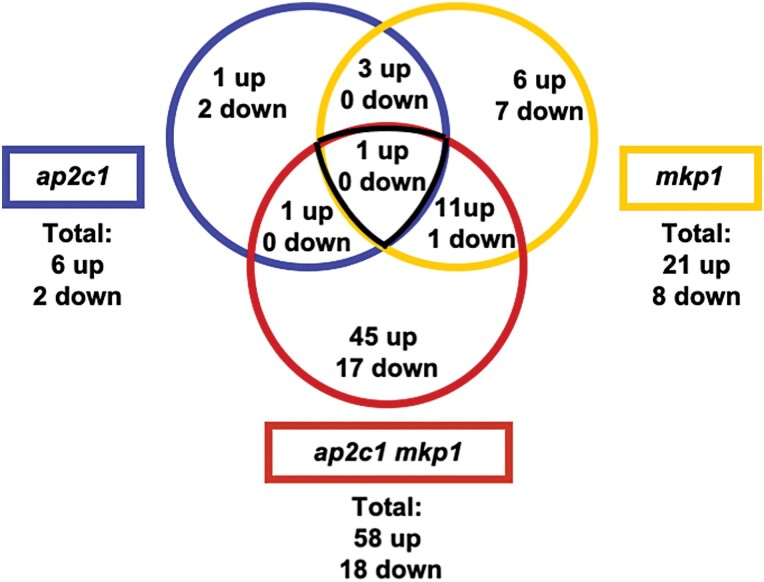
Venn diagram of TF genes differentially expressed in the MAPK phosphatase mutant plants. The number of genes up- or down-regulated at least 3-fold in *ap2c1*, *mkp1*, and *ap2c1 mkp1* plants compared with the WT in three biological replicates is indicated. The expression of 1880 TF-encoding genes was analysed.

**Fig. 5.**
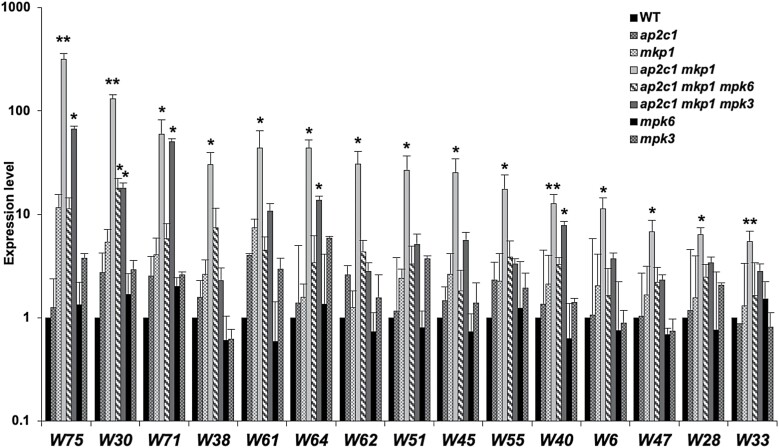
Expression of WRKY-encoding genes. The transcript levels of WRKY-encoding (W) genes were quantified by RT–qPCR in plants of the indicated genotypes and compared with the WT (expression levels in the WT were set to 1). Bars represent mean ±SD values of three replicates. Data are plotted on a log_10_ scale after normalization over WT values. ∗*P*<0.05, ∗∗*P*<0.01 (Mann–Whitney *U* test).

**Fig. 6.**
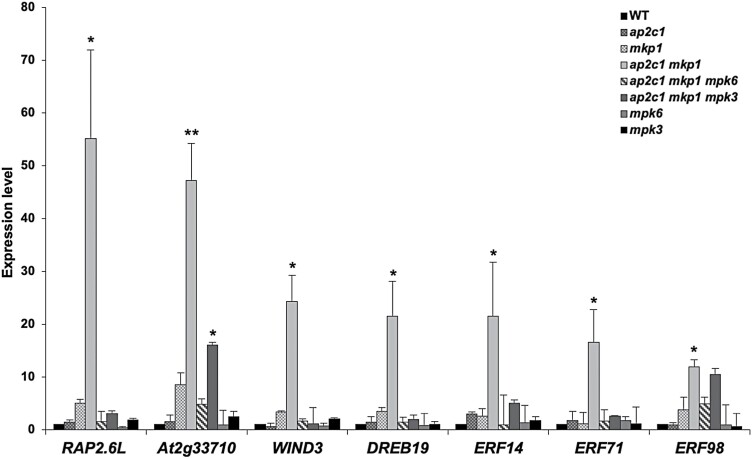
Genes encoding members of the AP2/ERF TF family are highly up-regulated in *ap2c1 mkp1* plants. Transcript levels of AP2/ERF-encoding genes were quantified by RT–qPCR in plants of the indicated genotypes and compared with the WT (expression levels in the WT were set to 1). Bars represent mean ±SD values of at least three replicates. ∗*P*<0.05, ∗∗*P*<0.01 (Mann–Whitney *U* test).

**Fig. 7.**
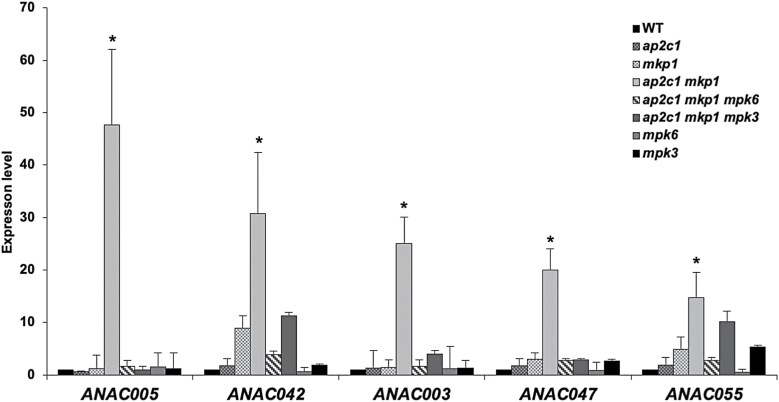
Genes encoding members of the ANAC TF family are highly up-regulated in *ap2c1 mkp1* plants. Transcript levels of *ANAC* genes were quantified by RT–qPCR in plants of the indicated genotypes and compared with the WT (expression levels in the WT were set to 1). Bars represent mean ±SD values of at least three replicates. ∗*P*<0.05 (Mann-Whitney *U* test).

To investigate if and how AP2C1 and MKP1 influence the regulation of gene expression under standard growth conditions, we used an RT–qPCR platform for high-throughput expression profiling of 1880 Arabidopsis TF-encoding genes (Balazadeh *et al.*, 2008). We selected genes showing an at least 3-fold mean difference of expression levels in *ap2c1*, *mkp1*, or *ap2c1 mkp1* plants compared with the WT. We identified three genes encoding TFs that were deregulated in *ap2c1* but not in *mkp1* (Supplementary Table S1), 25 genes that were deregulated in *mkp1* but not in *ap2c1* (Supplementary Table S2), and four genes concomitantly regulated by AP2C1 and MKP1 (Supplementary Table S3). Figure 4 shows the number of genes whose expression levels were changed in *ap2c1*, *mkp1*, or *ap2c1 mkp1* plants, compared with the WT. The TF genes dysregulated in the double mutant, and their expression values relative to the WT, are presented in Supplementary Table S4. The deregulation of 76 TF-encoding genes (58 up-regulated, 18 down-regulated) was found reproducibly in at least three different experiments in *ap2c1 mkp1* double mutant plants. Among them, genes encoding members of the WRKY family were most abundant: 15 *WRKY* genes were up-regulated (Fig. 5, Supplementary Table S4) and one was down-regulated (Supplementary Table S4). A further prevalent group of TF-encoding genes affected in *ap2c1 mkp1* plants includes *AP2*/*ERF* genes, described for their involvement in development, including *RAP2.6L* (Yang *et al.*, 2018) and *WIND3* (Smit *et al.*, 2020) (Fig. 6). ANAC TF family members are implicated in senescence and stress-related processes (Bu *et al.*, 2008; Jensen *et al.*, 2010; Wu *et al.*, 2012; Saga *et al.*, 2012). Our results show that several ANAC TF-encoding genes were up-regulated in *ap2c1 mkp1* plants (Fig. 7). Thus, our data suggest a cooperative function of AP2C1 and MKP1 in the transcriptional regulation of a set of *WRKY, AP2*/*ERF*, and *ANAC* genes in the WT.

Our observation that *ap2c1 mkp1 mpk6* and *ap2c1 mkp1 mpk3* plants are phenotypically much less affected than the *ap2c1 mkp1* double mutant suggested that severe phenotypic aberrations in the double mutant are mediated mainly by MPK6 and probably to a lesser extent by MPK3. This prompted us to investigate *ap2c1 mkp1 mpk6* and *ap2c1 mkp1 mpk3* plants for the expression of TFs misregulated in *ap2c1 mkp1*. Indeed, most of the TF genes strongly affected in *ap2c1 mkp1* plants (Supplementary Table S4) were not significantly altered in their expression in *ap2c1 mkp1 mpk6* and *ap2c1 mkp1 mpk3*, linking MAPK over-activation to the TF misexpression in the double mutant (Figs 5–7). Among these, the expression of *WRKY75*, *WRKY71*, *WRKY64*, and *WRKY40* (Fig. 5) and of *At2g33710* (Fig. 6) in the double mutant are more MPK3-independent, while the expression of other identified *WRKYs* (Fig. 5), the AP2/ERF family members *RAP2.6L*, *WIND3*, *DREB19*, *ERF14*, *ERF17, ERF71*, and *ERF98* (Fig. 6), and several ANAC TF-encoding genes, such as *ANAC005*, *ANAC042*, *ANAC003*, *ANAC047*, and *ANAC055* (Fig. 7), are mainly dependent on the presence of both MPK6 and MPK3.

### Defence responses, camalexin, SA, and the senescence marker gene *SAG12* are up-regulated in *ap2c1 mkp1* plants

It has been shown previously that *mkp1* plants accumulate higher levels of the phytoalexin camalexin (Bartels *et al.*, 2009). To investigate whether the expression of genes encoding camalexin biosynthesis enzymes was affected in *ap2c1 mkp1* plants, we studied the expression of a key gene in the pathway, *CYP71B15*/*PAD3*. A strong up-regulation (>300-fold) was detected in *ap2c1 mkp1* plants compared with the WT (Supplementary Fig. S6). Moreover, *ap2c1 mkp1 mpk6* plants still had remarkably high transcript levels (up-regulation >10-fold) of *CYP71B15*/*PAD3.* In addition, *mkp1* single mutant plants showed a ~10-fold up-regulation of the gene (Supplementary Fig. S6).

**Fig. 8.**
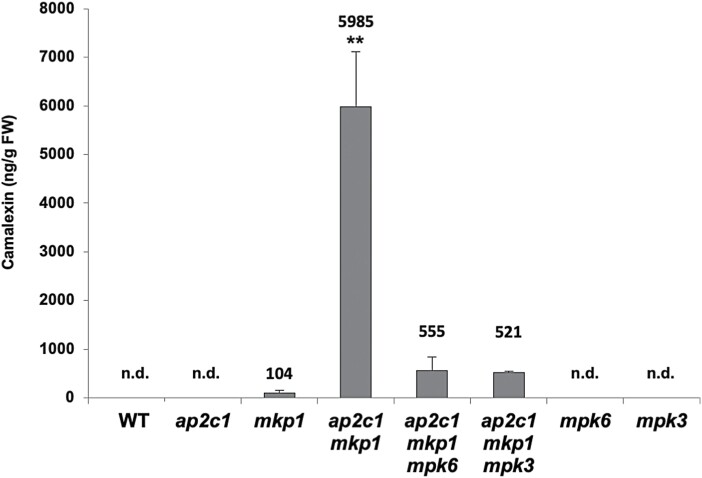
Camalexin accumulation in *ap2c1 mkp1* plants is mainly mediated by MPK6 and MPK3. Concentrations of total camalexin determined by HPLC in leaves of 4-week-old plants of the indicated genotypes. Results shown are means ±SE (*n*=4). n.d., Not detected. ∗∗*P*<0.01 (Student’s *t*-test).

To investigate whether the increased *CYP71B15*/*PAD3* expression level correlates with camalexin accumulation, total camalexin was quantified in WT and mutant plants. Indeed, we found increased camalexin concentrations in *mkp1* plants, in agreement with previous findings (Bartels *et al.*, 2009), and very high camalexin accumulation in the *ap2c1 mkp1* mutant, which was not solely dependent on MPK6 and MPK3 (Fig. 8).

**Fig. 9.**
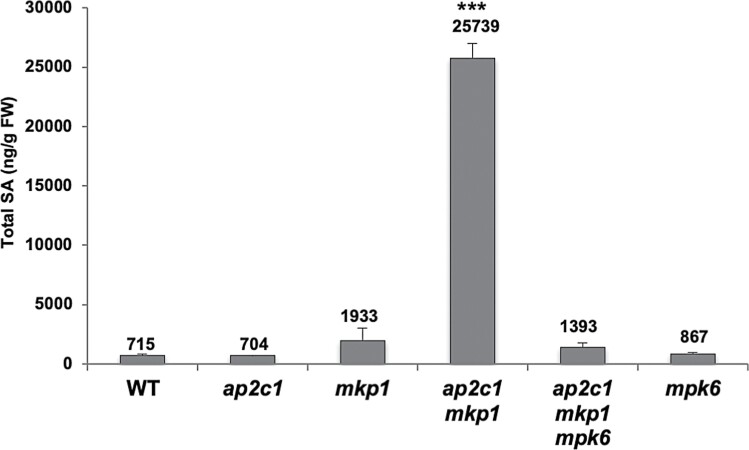
*ap2c1 mkp1* plants accumulate high concentrations of SA in an MPK6-dependent manner. Total SA concentrations of 5-week-old plants of the indicated genotypes grown under standard short-day conditions were determined by HPLC and expressed as ng g^–1^ FW. Error bars represent the SD of four biological replicates. ∗∗∗*P*<0.001 (Student’s *t*-test).

The up-regulation of MAPK activities and macroscopic lesion formation in leaves of *ap2c1 mkp1* indicated the possible activation of a hypersensitive-like response in these plants. Since this response is associated with the accumulation of the stress hormone SA, we measured SA in leaves of *ap2c1 mkp1* and *ap2c1 mkp1 mpk6*, as well as in WT and the single mutants. We found a 35-fold increase of SA in *ap2c1 mkp1* plants compared with the WT (Fig. 9), whereas *ap2c1*, *ap2c1 mkp1 mpk6*, and *mpk6* plants showed SA concentrations similar to the WT. In agreement with previous data (Bartels *et al.*, 2009), we also detected enhanced total SA concentrations (>2-fold) in *mkp1* plants compared with the WT (Fig. 9).

**Fig. 10.**
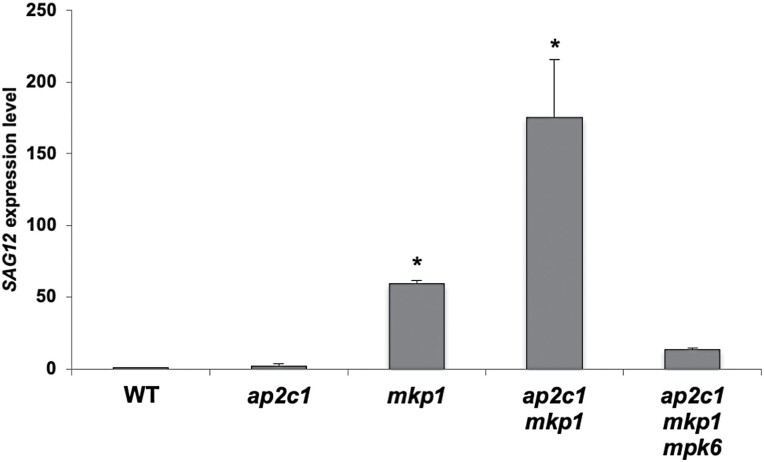
Up-regulation of the senescence-marker gene *SAG12* in *ap2c1 mkp1* plants is mainly mediated by MPK6. RT–qPCR quantification of *SAG12* transcript level in plants of the indicated genotypes compared with WT plants grown under standard short-day conditions. Error bars represent the SD of three biological replicates. ∗*P*<0.05 (Student’s *t*-test).

The leaf necrosis observed in *ap2c1 mkp1* leaves (Fig. 1A; Supplementary Fig. S1G) and the up-regulation of *WRKY6* (Fig. 5), which is a senescence-related marker gene (Rushton *et al.*, 2010), suggested that early senescence was induced in these plants. Thus, we investigated the expression of the senescence-specific marker gene *SENESCENCE-ASSOCIATED GENE12* (*SAG12*) (Noh and Amasino, 1999) and found that it was strongly up-regulated in *ap2c1 mkp1* plants, dependent on MPK6 (Fig. 10). These data, along with the up-regulation of *WRKY6* (Fig. 5), indicate aberrant early induction of senescence-related processes in the double phosphatase mutant.

## Discussion

### Coordinated control of MAPK activities by AP2C1 and MKP1

Acclimation for survival is a fundamental principle that relies on intracellular signalling in every organism. Different signals converge at the level of MAPK cascades and from there diverge into a range of different downstream pathways and responses (Andreasson and Ellis, 2010; Rodriguez *et al.*, 2010; Rasmussen *et al.*, 2012). Considering the broad spectrum of signals transduced by overlapping players of MAPK pathways, it is puzzling how response specificity is attained (Lampard *et al.*, 2009; Rodriguez *et al.*, 2010; Meng and Zhang, 2013). Several signalling scenarios have been investigated that could help explain pathway specificity, including activity-dependent kinase distribution and localization, protein complex formation (e.g. interaction with scaffolding proteins), and dephosphorylation by protein phosphatases (Krysan and Colcombet, 2018). Over the past decades, mainly the functions of MPK3/MPK4/MPK6 in diverse pathways have been described, indicating them as both points of divergence and integration hubs in cellular signalling (Peng *et al.*, 2018; Bigeard and Hirt, 2018).

Here, we provide evidence that two evolutionarily distinct MAPK phosphatases control stress-related signalling in Arabidopsis by inactivating an overlapping set of target MAPKs that mediate stress and defence responses. The Ser/Thr PP2C phosphatase AP2C1 and the DSP MKP1 contribute to ensure the appropriate inactivation of MAPKs during stress. Both AP2C1 and MKP1 target MPK3, MPK4, and MPK6 (Ulm *et al.*, 2002; Schweighofer *et al.*, 2007; Bartels *et al.*, 2009; Anderson *et al.*, 2011; Galletti *et al.*, 2011; Sidonskaya *et al.*, 2016; Shubchynskyy *et al.*, 2017). The enhanced activation of MAPKs by wounding and by PAMP (flg22), and the constitutive stress signalling in the absence of stress in *ap2c1 mkp1* plants, indicate that the lack of both MAPK phosphatases creates a shortfall downstream of MAPKs, exemplified by the deregulated expression of TF-encoding genes.

The increased kinase activity in *mkp1* plants versus WT at the earlier time points after wounding and flg22 application compared with *ap2c1* versus WT suggests that the contribution of MKP1 to inactivating MAPKs is already set before stress treatment, or during a very early stage of signalling. In agreement with this suggested function of MKP1 during early signalling, previous work has also shown that *mkp1* and *mkp1 ptp1* mutant plants demonstrate equally elevated MAPK activities without stress treatment, underlining the major role of MKP1 in MAPK regulation in ambient conditions (Bartels *et al.*, 2009). On the contrary, AP2C1 adds to MAPK inactivation at later time points. It is possible that AP2C1 is primarily responsible for keeping the stress-induced activation below a certain threshold and controlling the duration of kinase activation during acute stress, acting as an ‘emergency brake’, while MKP1 is predominantly responsible for suppressing kinase activities under normal conditions, providing a ‘constitutive brake’. This hypothesis is supported by the induction of *AP2C1* expression by a plethora of stresses, while *MKP1* shows comparatively marginal changes in expression (https://www.genevestigator.com). These observations are also consistent with a recent comprehensive analysis of the Arabidopsis proteome, which covers more than 14 000 proteins and where in ambient conditions the overall MKP1 abundance exceeds by far that of AP2C1 (http://athena.proteomics.wzw.tum.de/) (Mergner *et al.*, 2020), underlining the rather specific role of AP2C1 under stress conditions. The AP2C1 paralogues AP2C2 and AP2C3 (Umbrasaite *et al.*, 2010; Umbrasaite *et al.*, 2011; Schweighofer *et al.*, 2014) as well as MKP1 and PTP1 interact with the same MAPKs and dephosphorylate them to various extents (Bartels *et al.*, 2009). However, the rather mild phenotypes of *ap2c2 mkp1* and *ap2c3 mkp1* plants, and the WT-like appearance of *ap2c1 ptp1* (Fig. 1C) compared with *ap2c1 mkp1* plants, clearly indicate specific genetic interactions and redundant functions of the evolutionarily distant AP2C1 and MKP1 phosphatases in the regulation of signalling pathways.

### Manifestation of cell death in *ap2c1 mkp1* plants

The lesions in leaves of *ap2c1 mkp1* plants suggest autoimmune-like responses, most likely caused by misregulation of MAPKs and/or failed control of guarding resistance (R) proteins (Rodriguez *et al.*, 2016). AP2C1 and MKP1 share the target MAPKs MPK3, MPK4, and MPK6, where MPK4 and some of its upstream MAPK cascade members, for example, MEKK1 and MKK1/2, were originally described as negative regulators of plant immunity based on their mutant plant phenotypes (Petersen *et al.*, 2000; Rasmussen *et al.*, 2012). The improper activation of the *R*-gene *SUMM2* is mainly responsible for the phenotypic defects of the *mpk4* mutant and other mutant plants in the pathway, identifying the MEKK1-MKK1/2-MPK4 module as a positive regulator of stress responses (Zhang *et al.*, 2012). Similar observations connecting phosphatase-targeted MAPKs with autoimmune-like phenotypes have been made by ectopically expressing constitutively active MPK3 (Genot *et al.*, 2017) or by inducibly expressing MKK5, which activates MPK3 and MPK6 (Lassowskat *et al.*, 2014). Both approaches led to a plethora of phenotypic and molecular changes, including dwarfism, lesion formation, de-repression of defence gene expression, and the accumulation of stress hormones, similar to the *ap2c1 mkp1*-related phenotypes described in this work (Figs 1–10; Supplementary Figs S1, S3, S4, S6; Supplementary Table S4).

The single *mkp1* and double *mkp1 ptp1* mutants show constitutive defence responses including increased levels of SA and camalexin, suggesting partially overlapping functions of MKP1 and PTP1 in repressing SA biosynthesis (Bartels *et al.*, 2009). Similarly, the strong accumulation of SA and camalexin in *ap2c1 mkp1* compared with *mkp1* plants suggests a collaborative action of both AP2C1 and MKP1 as negative regulators of SA and camalexin production (Figs 8, 9; Supplementary Fig. S6). The camalexin accumulation in *ap2c1 mkp1* is largely reduced in *ap2c1 mkp1 mpk6* and *ap2c1 mkp1 mpk3* triple mutants, indicating the dependency of camalexin biosynthesis on partially redundant actions of MPK6 and MPK3, which is in agreement with previous findings (Mao *et al.*, 2011). The SA accumulation in *ap2c1 mkp1* plants is probably mainly MPK6-dependent, as the introduction of the *mpk6* mutation in *ap2c1 mkp1 mpk6* plants restores SA concentrations similar to those of WT and *mkp1 ptp1 mpk6* mutant plants (Bartels *et al.*, 2009). Notably, rescue of the severe *ap2c1 mkp1* growth phenotypes by elevated temperature is in accordance with the observed temperature dependency of SA-related phenotypes (Ichimura *et al.*, 2006; Suarez-Rodriguez *et al.*, 2007; Su *et al.*, 2007), as well as with the suppression of *SNC1* expression and reduction of SNC1 activity by high temperature (Yang and Hua, 2004; Zhu *et al.*, 2010). The resistance protein SNC1 is a modifier of *mkp1* in the Col-0 accession, where partial rescue of *mkp1* and *mkp1 ptp1* growth phenotypes by a loss-of-function *snc1* mutation indicates a sensitized SNC1 signalling pathway in the absence of MKP1 (Bartels *et al.*, 2009).

Previous findings that SA acts together with ET to regulate cell death (Rao *et al.*, 2002), the requirement of ET biosynthesis for H_2_O_2_ accumulation and subsequent cell death (Overmyer *et al.*, 2003), and the induction of cell death in Arabidopsis leaves by persistent activation of MAPKs with gain-of-function MKK4 and MKK5 (Ren *et al.*, 2002) all correlate with the cell-death phenotype observed in the *ap2c1 mkp1* mutant, where MAPKs—and other stress-related factors—may be (hyper)-activated. Therefore, we conclude that the majority of the phenotypes observed in *ap2c1 mkp1* plants, both visible and molecular, are due to the misregulation of MAPK pathways, even in the absence of stress.

### AP2C1 and MKP1 affect MAPK-regulated ET biosynthesis

Activated MPK6 controls ET levels by inducing the transcription of *ACS* family genes and by phosphorylating ACS proteins, the rate-limiting enzymes in ET biosynthesis. Phosphorylated ACSs become more stable and, thus, ET synthesis is increased by elevated MPK6 activity (Kim *et al.*, 2003; Liu and Zhang, 2004; Xu *et al.*, 2008; Li *et al.*, 2012). In *ap2c1 mkp1*, the increased ET production is likely due to, at least in part, the highly increased expression of *ACS6* compared with the WT. A considerable additive effect on ET overproduction in the double *ap2c1 mkp1* mutant compared with the *mkp1* single mutant suggests that even though MKP1 is a determining MAPK phosphatase affecting ET production, there are overlapping and non-redundant functions of AP2C1 and MKP1 in the regulation of stress-induced ET biosynthesis. The detection of increased and MPK6-dependent expression of *WRKY33*, which encodes a TF that binds to the promoter of *ACS* genes and is a substrate of MPK3 and MPK6, suggests an involvement of WKRY33 itself in *ACS* overexpression in *ap2c1 mkp1* plants (Figs 3, 5; Li *et al.*, 2012). The identification of genes encoding TFs of the AP2/ERF family (ET-responsive element-binding proteins) among the most strongly induced ones in *ap2c1 mkp1* plants suggests a path to increased ET amounts in these plants.

### AP2C1 and MKP1 control the expression of stress-responsive TF-encoding genes, predominantly via partially redundant actions of MPK6 and MPK3

Transcriptional reprogramming in response to activated MAPK signalling suggests an involvement of TFs. Our results indicate that the concomitant lack of the MAPK regulators AP2C1 and MKP1 results in elevated basal MAPK activities and leads to highly increased expression of *WRKY* TF genes, in some cases by more than 100-fold compared with the WT. The *ap2c1 mkp1* mutant phenotypes and the described functions of some up-regulated *WRKY*s indicate that stress responses are constitutively active in these plants. This correlates with reports demonstrating an involvement of WRKYs in oxidative stress responses, in the induction of ET and camalexin biosynthesis (*WRKY30, WRKY33*), the response to pathogens (*WRKY71, WRKY40*), basal defence (*WRKY38*), and defence- and senescence-related processes (*WRKY6*) (Rushton *et al.*, 2010).

Direct feedback mechanisms among WRKYs themselves have been demonstrated (Mao *et al.*, 2011) and are generally proposed, where WRKYs positively auto-regulate their own gene expression and/or cross-regulate the expression of other *WRKY* genes (Pandey and Somssich, 2009; Mao *et al.*, 2011; Birkenbihl *et al.*, 2017). Thus, it could be that the increased activation of MAPKs in *ap2c1 mkp1* plants leads to phosphorylation and thus activation of MAPK target WRKY proteins, which serve as activated TFs for a further series of *WRKY* genes. In any case, MPK6 and MPK3 seem to be major players responsible for mediating the up-regulation of several *WRKY*s, *AP2*/*ERF*s, *ANAC*s, and other TF-encoding genes. Both MAPKs control the expression of several *WRKY*s to different extents, as shown in *ap2c1 mkp1* plants compared with *ap2c1 mkp1 mpk6* and *ap2c1 mkp1 mpk3* plants (Fig. 5). These data also demonstrate that not only MAPKs but also other factor(s) affect *WRKY* gene expression. We confirmed MPK6- and MPK3-dependent *WRKY33* expression (Mao *et al.*, 2011); however, the higher MPK4 activities in *ap2c1 mkp1* may also lead to higher amounts of active WRKY33 protein (Qiu *et al.*, 2008; Birkenbihl *et al.*, 2017). Our data suggest that AP2C1 and MKP1 may play a dual role in regulating camalexin biosynthesis, on the one hand by controlling MPK6 and MPK3 activities, which positively regulate *WKRY33* expression, and on the other hand by controlling MPK4 activity, which in turn stimulates WRKY33, leading to the transactivation of *CYP71B15/PAD3*.

Remarkably, the concomitant absence of AP2C1 and MKP1 in non-stress conditions causes a distinct transcriptional activation of TFs compared with single mutant *ap2c1* and *mkp1* plants after stress treatments: challenging *ap2c1* plants with *Pto* for 4 h led to 88 differentially regulated TFs (Shubchynskyy *et al.*, 2017), whereas only four TFs from this set were up-regulated in untreated *ap2c1 mkp1* plants (Supplementary Table S4).

Comparing transcriptional changes of PAMP-treated *mkp1* plants (Jiang *et al.*, 2017) with untreated *ap2c1 mkp1* double mutant plants (Supplementary Table S4) revealed a more MKP1-specific TF induction: a next-generation sequencing (RNA-seq) transcriptome analysis of *mkp1* plants 90 min after treatment with the PAMP elf26 revealed that from 67 identified TFs among the 1102 MKP1-dependent transcripts (Jiang *et al.*, 2017) 21 TFs were also changed in untreated *ap2c1 mkp1* plants (Supplementary Table S4). In accordance with the severe phenotype of *mkp1 ptp1* mutant plants and its suppression by elevated growth temperature or crossing with *mpk3* and *mpk6* mutants (Bartels *et al.*, 2009), the proposed predominant role of MKP1 under non-stress conditions and AP2C1 during stress conditions to regulate (MAPK) signalling are emphasized.

A PAMP flg22-activated MPK3/MPK6 pathway was previously reported to elevate *WRKY22* and *WRKY29* expression (Asai *et al.*, 2002). Strongly enhanced MPK3/MPK6 activities, but unaffected expression of either *WRKY22* or *WRKY29* in untreated *ap2c1 mkp1* plants, after PAMP elf26 and *Pto* treatment (Jiang *et al.*, 2017; Shubchynskyy *et al.*, 2017) show that for *WRKY22*/*29* overexpression, MPK3 and MPK6 hyperactivation is necessary (Asai *et al.*, 2002) but not sufficient (Fig. 5; Supplementary Table S4), and that other factors (possibly MAPKs) may be playing a role instead of MPK3 and MPK6.

### Senescence is repressed by AP2C1 and MKP1 phosphatases in an MPK6-dependent way

Several lines of evidence indicate that the *ap2c1 mkp1* mutant undergoes precocious senescence. Leaf senescence is a highly regulated process that finally leads to cell death and tissue disintegration, at the same time contributing to the fitness of the whole plant. Senescence is controlled by endogenous and environmental cues, and can be triggered prematurely by different abiotic/biotic stresses such as pathogen attack, wounding, UV light irradiation, or high ozone levels (Hanfrey *et al.*, 1996; Miller *et al.*, 1999; John *et al.*, 2001; He *et al.*, 2001; Lim *et al.*, 2007). The MKK9-MPK6 cascade has been shown to positively regulate leaf senescence in Arabidopsis (Zhou *et al.*, 2009). Hyperactivation of MPK6 and other MAPKs, in addition to autoimmune-like responses, also promotes senescence, which is very evident in older leaves of *ap2c1 mkp1* plants and correlates with significant up-regulation of the senescence-specific marker gene *SAG12* (Noh and Amasino, 1999; Guo and Gan, 2005). Partial suppression of *SAG12* overexpression in *ap2c1 mkp1 mpk6* suggests an MPK6-dependent regulation (possibly involving other MAPKs) in promoting plant senescence.

Genome-wide transcriptomics previously identified several senescence-related TFs from the ANAC family (Breeze *et al.*, 2011). We identified strong MAPK-dependent induction of *ANAC005*, *JUB1*/*ANAC042* (Wu *et al.*, 2012; Saga *et al.*, 2012; Shahnejat-Bushehri *et al.*, 2016), *ANAC003*/*XVP* (Yang *et al.*, 2020), *ANAC047* (Mito *et al.*, 2011), and *ANAC055* (Tran *et al.*, 2004; Bu *et al.*, 2008; Hickman *et al.*, 2013; Schweizer *et al.*, 2013) in *ap2c1 mkp1* plants. This induction of senescence-related TFs reveals a novel link between senescence-related processes and MAPK signalling.

We conclude that the induction of senescence processes as well as hypersensitive response-like cell death results in the premature death of leaves in *ap2c1 mkp1* plants. The crosstalk between senescence and abiotic stress or pathogen responses is accentuated in *ap2c1 mkp1* plants, where up-regulation of TFs involved in these processes occurs.

Taken together, our results show that two evolutionarily unrelated MAPK phosphatases, AP2C1 and MKP1, perform both distinct and overlapping functions in the regulation of stress-induced MPK3, MPK4, and MPK6 activities. Our genetic dissection indicates that the known roles of MPK6 and MPK3 in mediating cell death and ET-, SA- and senescence-related phenotypes are counter-balanced by both AP2C1 and MKP1. It also demonstrates that the expression of specific TF-encoding genes is affected by hyperactivation of MAPK(s) due to the lack of these two MAPK phosphatases *in planta*, revealing potential new signalling target genes downstream of MPK6 and MPK3. In the future, the study of individual and combinatorial mutants will allow us to genetically disentangle the contribution of specific protein kinases and phosphatases to complex signalling networks and downstream cell responses.

## Supplementary data

The following supplementary data are available at *JXB* online.

Fig. S1. Loss of both AP2C1 and MKP1 leads to severe phenotypes in growth and development.

Fig. S2. Plant phenotypes grown in conditions with increased humidity and elevated temperature.

Fig. S3. Phenotypes of Arabidopsis single, double and triple mutant plants.

Fig. S4. AP2C1 and MKP1 control stress-induced MAPK activities.

Fig. S5. Detection of *MKP1* and *AP2C1* expression levels in *ap2c1* and *mkp1* mutants.

Fig. S6. Up-regulation of the camalexin biosynthetic gene *CYP71B15/PAD3* is mainly mediated by MPK6.

Table S1. Expression of TF-encoding genes modulated by the absence of AP2C1 but not MKP1.

Table S2. Expression of TF-encoding genes modulated by the absence of MKP1 but not of AP2C1.

Table S3. Expression of TF-encoding genes modulated by the absence of both MKP1 and AP2C1.

Table S4. TF-encoding genes deregulated in *ap2c1 mkp1* plants.

Table S5. The Arabidopsis Genome Initiative (AGI) codes of the genes analysed in this report.

erac018_suppl_Supplementary_Figures_S1-S6_Tables_S1-S5

## Data Availability

The data supporting the findings of this study are available from the corresponding author, Alois Schweighofer, upon request.
