# Supplementary material for: Dual control of MAPK activities by AP2C1 and MKP1 MAPK phosphatases regulates defence responses in Arabidopsis
Source: J Exp Bot. 2022 Jan 28;73(8):2369–84. doi: 10.1093/jxb/erac018 (PMC9015810; doi:10.1093/jxb/erac018)
Supplement: erac018_suppl_Supplementary_Figures_S1-S6_Tables_S1-S5 [file erac018_suppl_supplementary_figures_s1-s6_tables_s1-s5.pdf]

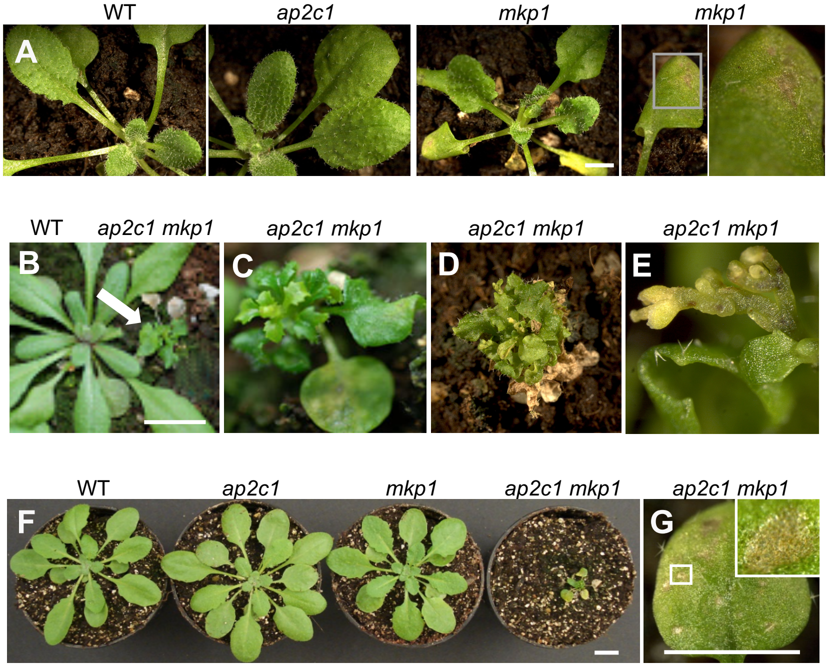

Supplementary Fig. S1. Loss of both AP2C1 and MKP1 leads to severe phenotypes in growth and development.

(A) Phenotypes of WT, *ap2c1*, and *mkp1* plants grown for three weeks in long-day conditions. Scale bars = 0.5 cm.

(B) Phenotypes of WT (left) and *ap2c1 mkp1* (right, indicated by arrow) grown for 3.5 weeks in standard long-day conditions. During the first 18 days of growth, the phenotypic differences of *ap2c1 mkp1* compared to WT plants are visibly manifested as a difference in plant size while after approximately 3.5 weeks (26 days) premature death of leaf tissue as well as abnormal leaf growth and morphology of *ap2c1 mkp1* plants (right, indicated by arrow) become apparent. Scale bar = 1 cm.

(C) Close-up of *ap2c1 mkp1* plant grown for 3.5 weeks in standard long-day condition.

(D) Phenotype of seven-week-old *ap2c1 mkp1* plant grown in standard long-day condition.

(E) Close-up of seven-week-old *ap2c1 mkp1* plant showing misshaped inflorescence.

(F) Phenotypes of WT, *ap2c1*, *mkp1* and *ap2c1 mkp1* plants grown for 8 weeks in short-day conditions. Scale bars = 1 cm.

(G) Formation of macroscopic lesions in leaves of *ap2c1 mkp1* plant shown in Supplemental Fig. 1F. Scale bars = 0.5 cm.

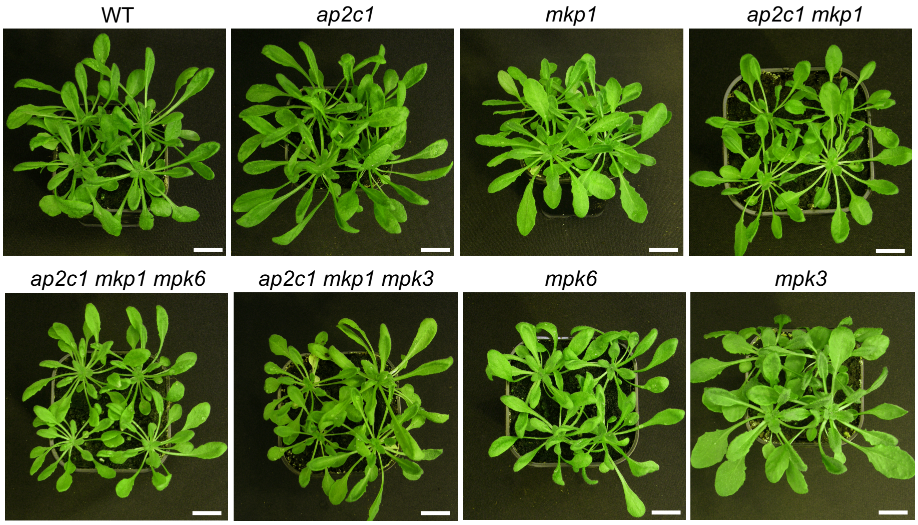

Supplemental Fig. S2. Plant phenotypes grown in conditions with increased humidity and elevated temperature.
Phenotypes of five-week-old plants of the indicated genotypes grown in a tray with closed lid in short day conditions at 24ºC and saturating relative humidity. Scale bars = 1 cm.

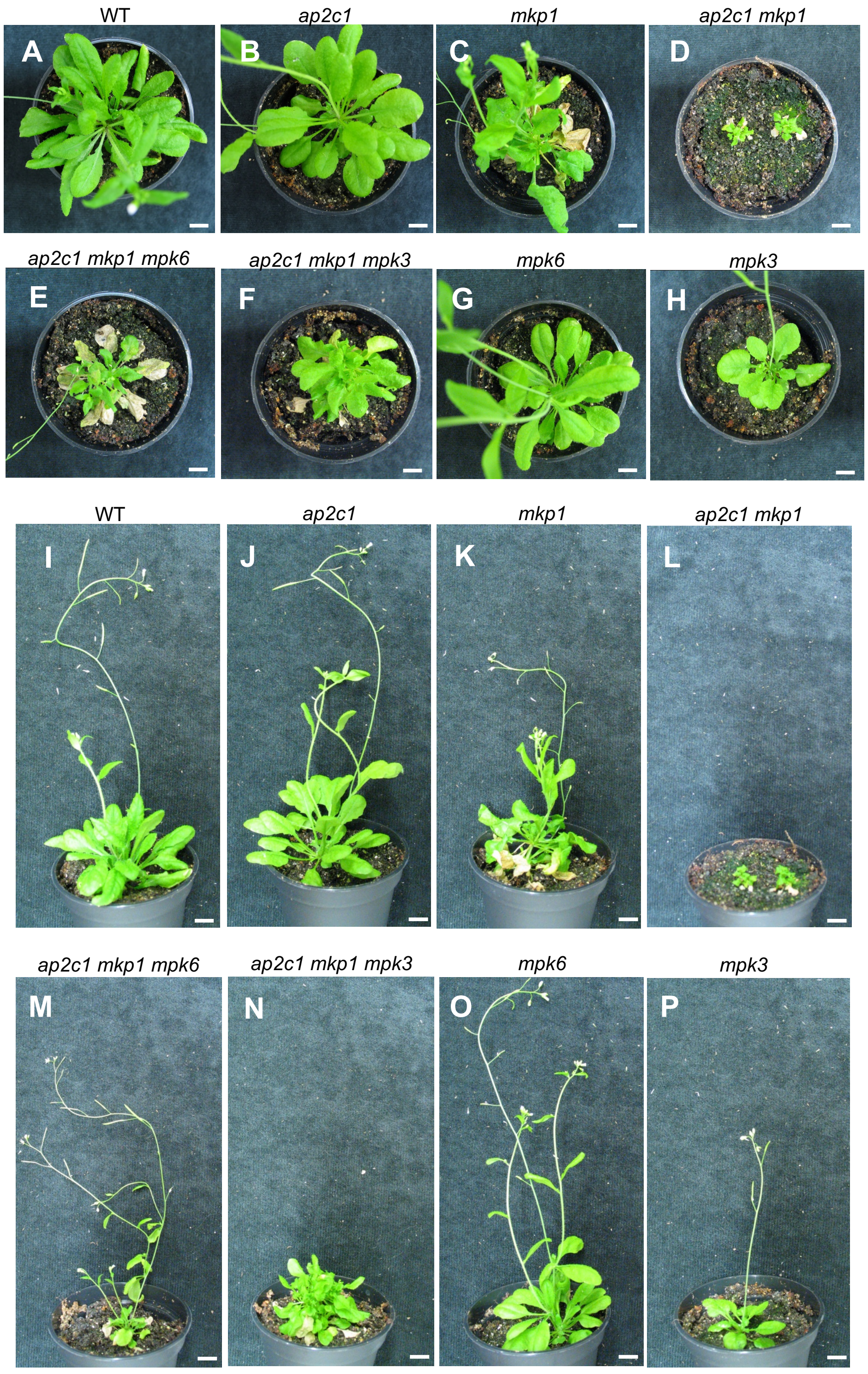

Supplementary Fig. S3. Phenotypes of Arabidopsis single, double and triple mutant plants.
(A – H) Phenotypes of plants of the indicated genotypes grown for four weeks in short-day conditions, followed by three weeks in long-day conditions. Scale bars = 1 cm.
(I – P) Phenotypes of eight-week-old plants of the indicated genotypes grown for the first six weeks in short-day and for a further two weeks in long-day conditions. The *ap2c1 mkp1* double mutant displays a severe dwarf phenotype, premature leaf decay, lack of normal shoot development, and strongly impaired inflorescence growth. These phenotypes were mainly rescued in *ap2c1 mkp1 mpk6* mutant plants, and partially in *ap2c1 mkp1 mpk3* mutant plants. Scale bars = 1 cm.

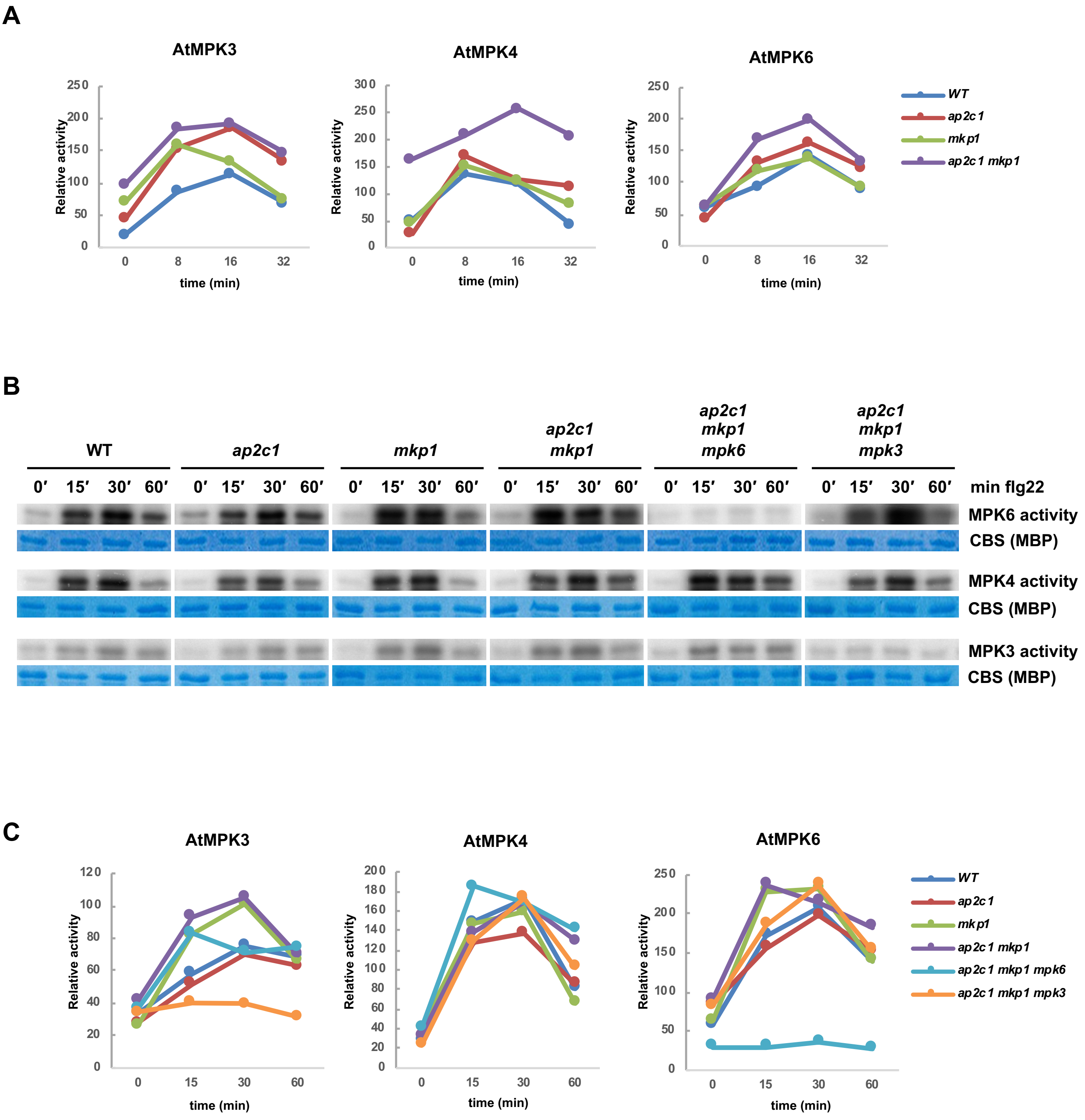

Supplementary Fig. S4. AP2C1 and MKP1 control stress-induced MAPK activities.

(A) Quantification of wound-induced MAPK activities as shown in Fig. 2. The radioactive signals were captured by a phosphorimaging plate, scanned with a laser-based scanner (Typhoon) and signal intensities determined using ImaqeQuant software. Time (min): minutes after wounding; relative activity: obtained values were divided by factor 10000.

(B) Analysis of flg22-induced MPK6, MPK4 and MPK3 kinase activities of leaves from five-week-old plants of the indicated genotypes grown in short-day conditions. MAPK activities were determined after immunoprecipitation by phosphorylation of myelin basic protein (MBP) detected by autoradiography. The entire kinase assay is based on one common master mix containing MBP and γ-ATP. Loading is demonstrated by Coomassie Blue staining (CBS); min flg22: minutes after treatment with 100 nM flg22.

(C) Quantification of flg22-induced MAPK activities shown in Supplementary Fig. 4B. The radioactive signals were captured by a phosphorimaging plate, scanned with a laser-based scanner (Typhoon) and signal intensities determined using ImaqeQuant software. Time (min): minutes after flg22 treatment; relative activity: obtained values were divided by factor 10000.

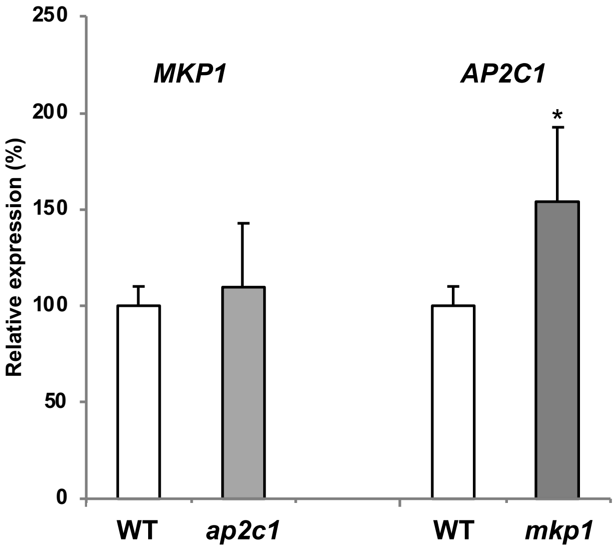

Supplementary Fig. S5. Detection of *MKP1* and *AP2C1* expression levels in *ap2c1* and *mkp1* mutants.

Levels of *MKP1* and *AP2C1* transcripts were quantified by RT-qPCR in 14-day-old seedlings grown in long-day conditions, and compared with levels in WT plants. Transcript amounts of *MKP1* and *AP2C1* in WT were taken as 100%. The relative transcript amounts were normalized to the reference gene, *ACTIN2*. Error bars indicate SD. Results are the mean of two biological and two technical replicates for each experiment, *p < 0.05 Student´s *t*-test.

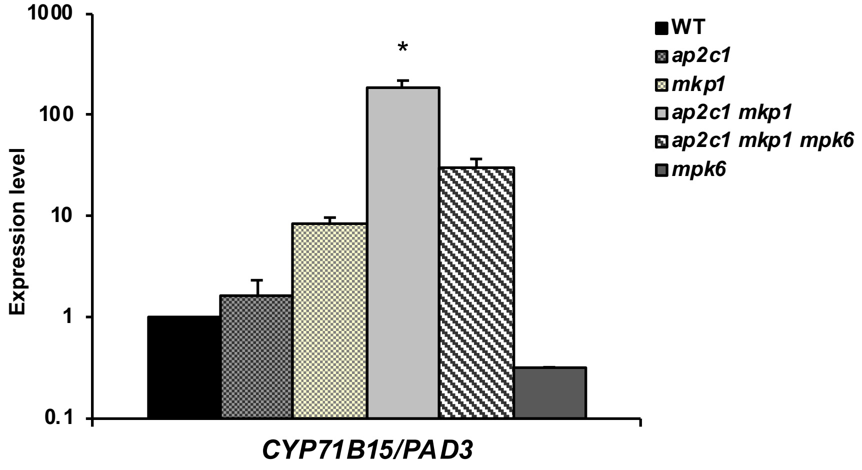

Supplementary Fig. S6. Upregulation of the camalexin biosynthetic gene *CYP71B15*/*PAD3* is mainly mediated by MPK6.

Transcript level of *CYP71B15*/*PAD3*, an enzyme required for camalexin biosynthesis, was quantified by RT-qPCR in plants of the indicated genotypes and compared to WT, where expression levels were set to 1. Bars represent mean values of at least three replicates ± SD, expressed on a $\log_{10}$ scale. *$p < 0.05$, Student's *t*-test.

Supplementary Table S1. Expression of TF-encoding genes modulated by the absence of AP2C1 but not of MKP1. Transcript levels were quantified by RT-qPCR and expressed as log_2 of fold change (FC) for each mutant compared to WT. Genes affected by at least 1.56-fold only in *ap2c1* while not more than 0.9-fold in *mkp1* are listed. Data are from two independent biological replicates and are reported with SE.

AGI	gene family	gene name(s)	***ap2c1*** log_2 FC	SE of -ΔΔct	***mkp1*** log_2 FC	SE of -ΔΔct
At2g46130	WRKY	WRKY43	**1.56**	0.40	-0.46	0.77
At1g65130	C2H2		**-1.90**	0.13	-0.42	0.29
At3g27810	MYB	MYB21/MYB3	**-3.79**	0.32	-0.27	0.35

Supplementary Table S2. Expression of TF-encoding genes modulated by the absence of MKP1 but not of AP2C1. Transcript levels were quantified by RT-qPCR and are expressed log_2 of fold change (FC) for each mutant compared to WT. Genes affected by at least 1.56-fold only in *mkp1* while not more than 0.9-fold in *ap2c1* are listed. Data are from two independent biological replicates and are reported with SE.

AGI	gene family	gene name(s)	***ap2c1*** log_2 FC	SE of -ΔΔct	***mkp1*** log_2 FC	SE of -ΔΔct
At5g43650	bHLH	BHLH92	-0.19	0.75	**3.77**	0.27
At1g71520	AP2/EREBP		0.41	0.91	**3.71**	0.54
At5g13080	WRKY	WRKY75	0.61	0.13	**3.21**	0.09
At3g49760	bZIP	BZIP5	-0.40	0.69	**2.49**	0.20
At2g43000	NAC	ANAC042/JUB1	0.61	0.04	**2.36**	0.29
At2g33710	AP2/EREBP		0.34	0.10	**2.29**	0.35
At5g56960	bHLH		-0.14	0.25	**2.29**	0.15
At2g47190	MYB	MYB2	0.48	0.11	**2.23**	0.30
At1g66600	WRKY	WRKY63	-0.60	0.25	**2.13**	0.70
At5g13330	AP2/EREBP	RAP2.6L	0.63	0.05	**2.08**	0.16
At1g48150	MADS	AgL74	0.76	0.09	**1.93**	0.17
At2g22770	bHLH	NAI	0.56	0.48	**1.81**	0.75
At3g23230	AP2/EREBP	ERF98	-0.16	0.08	**1.78**	0.76
At2g38340	AP2/EREBP	DREB19	0.31	0.13	**1.75**	0.13
At5g23000	MYB	MYB37	0.87	0.33	**1.69**	0.46
At1g62975	bHLH		0.12	0.45	**1.62**	0.40
At1g25470	AP2/EREBP	CRF12	0.41	0.89	**1.58**	0.14
At2g27220	HB	BLH5	-0.47	0.14	**-1.57**	0.69
At2g25820	AP2/EREBP	ESE2	-0.21	0.20	**-1.65**	0.22
At3g58070	C2H2	glS	-0.46	0.41	**-1.87**	0.12
At3g46130	MYB	MYB48	-0.23	0.19	**-1.97**	0.22
At3g15320	MYB		-0.70	0.05	**-2.00**	0.64
At3g53680	PHD finger		-0.41	0.04	**-2.06**	0.15
At5g51870	MADS	AgL71	-0.68	0.14	**-2.09**	0.20
At4g22950	MADS	AgL19	0.58	0.63	**-2.48**	0.11

Supplementary Table S3. Expression of TF-encoding genes modulated by the absence of both MKP1 and AP2C1. Transcript levels were quantified by RT-qPCR and are expressed as fold change (FC) in log_2 scale for each of the mutants compared to WT. Genes affected by at least 1.56-fold both in *ap2c1* and *mkp1* are listed. Data are from two independent biological replicates and are reported with SE.

AGI	gene family	gene name(s)	***ap2c1*** log_2 FC	SE of -ΔΔct	***mkp1*** log_2 FC	SE of -ΔΔct
At3g47870	AS2 (LOB) I	ASL29/LBD27/SCP	**2.24**	0.25	**3.57**	0.07
At2g13150	bZIP	BZIP31	**2.25**	0.77	**3.76**	0.76
At1g03790	C3H	SOM/TZF4	**2.27**	0.15	**1.81**	0.49
At1g18860	WRKY	WRKY61	**1.99**	0.11	**2.49**	0.08

Supplementary Table S4. TF-encoding genes deregulated in *ap2c1 mkp1* plants.
Gene transcript levels were quantified by RT-qPCR and expressed as fold change in log_2 scale for each of the mutants compared to WT. Genes affected in *ap2c1 mkp1* compared to WT by at least 1.56-fold in log_2 scale are listed. Data are from three independent biological replicates and are reported with SD.

**upregulated in *ap2c1 mkp1***								
			ap2c1		***mkp1***		***ap2c1 mkp1***	
**AGI**	**family**	**gene name(s)**	**-ΔΔct**	**SD**	**-ΔΔct**	**SD**	**-ΔΔct**	**SD**
At5g13330	AP2/EREBP	RAP2.6L	0,53	0,15	2,33	0,37	5,79	1,77
At1g36060	AP2/EREBP	WIND3	-0,60	0,50	1,77	0,09	4,60	0,95
At3g23240	AP2/EREBP	ERF1	1,02	0,11	2,65	0,20	4,44	1,85
At2g38340	AP2/EREBP	DREB19	0,54	0,37	1,81	0,39	4,43	1,35
At1g04370	AP2/EREBP	ERF14/ERF97	1,57	0,24	1,35	0,77	4,43	2,11
At2g47520	AP2/EREBP	ERF71/HRE2	0,78	0,81	0,22	0,41	4,06	1,50
At5g64750	AP2/EREBP	ABR1	0,46	0,91	1,13	0,72	2,38	0,33
At2g33710	AP2/EREBP		0,59	0,50	3,09	0,84	5,56	0,83
At1g71520	AP2/EREBP		0,41	0,91	3,71	0,54	5,05	1,33
At3g23230	AP2/EREBP	ERF98	-0,16	0,08	1,91	1,24	3,58	0,40
At2g40470	AS2 (LOB) I	LBD15	0,31	0,94	1,72	0,51	4,30	1,10
At1g67100	AS2 (LOB) II	LBD40	0,79	1,00	1,54	0,37	5,91	1,92
At5g43650	bHLH	BHLH92	-0,03	0,65	2,22	2,20	5,88	1,94
At2g43140	bHLH	BHLH129	0,17	0,14	1,78	0,24	4,02	0,79
At5g56960	bHLH		-0,14	0,25	1,94	1,21	5,62	0,81
At1g08320	bZIP	BZIP21/TGA9	0,93	0,64	1,87	0,25	4,15	1,14
At3g53600	C2H2	ZAT18	0,59	1,13	1,38	1,84	4,58	2,56
At5g67450	C2H2	AZF1	-0,12	0,48	0,64	0,35	3,33	1,15
At5g04390	C2H2		0,15	0,62	-0,22	0,93	2,88	0,82
At2g37430	C2H2	ZAT11	0,21	0,66	0,33	0,73	2,68	0,27
At3g46080	C2H2		0,21	0,65	0,80	0,57	7,15	1,26
At3g46090	C2H2	ZAT7	-0,77	0,40	0,16	0,55	5,92	0,92
At5g59820	C2H2	ZAT12/RHL41	0,54	1,14	0,50	0,84	4,14	0,85
At1g13300	GARP-G2-like	HRS1/NIGT1.4	0,38	1,09	0,46	1,17	4,50	0,96
At5g19520	HB	MSL9	0,91	0,91	0,02	1,56	2,47	0,38
At4g36990	HSF	HSFB1/HSF4/TBF1	0,63	0,44	0,31	1,13	4,62	1,21
At4g18870	HSF		3,27	0,83	1,24	1,10	7,52	2,84
At5g20240	MADS		0,02	0,88	0,56	0,95	4,86	1,06
At5g23000	MYB	MYB37/RAX1	0,87	0,33	1,77	0,40	4,26	1,44
At1g18570	MYB	MYB51/HIG1	0,21	0,50	0,60	0,54	2,62	0,70
At1g74080	MYB	MYB122	0,21	0,58	0,61	1,06	8,00	1,78
At3g23250	MYB	MYB15	0,93	0,94	0,45	0,72	4,51	1,75
At1g02250	NAC	ANAC005	-0,68	0,15	0,23	0,51	5,57	1,69
At1g02220	NAC	ANAC003	0,34	0,90	0,52	0,50	4,65	0,93
At3g04070	NAC	ANAC047	0,84	0,62	1,57	0,67	4,32	0,86
At3g15500	NAC	ANAC055	0,90	0,70	2,28	1,11	3,88	1,29
At1g01010	NAC	ANAC001	-0,03	0,38	-0,02	0,62	3,12	0,63
At3g44350	NAC	ANAC061	-0,41	0,62	-0,78	0,59	3,09	1,05
At2g43000	NAC	ANAC042	0,78	0,62	3,16	0,83	4,94	1,89
At2g38250	Trihelix		-0,01	0,68	0,19	0,69	3,13	0,93
At3g01970	WRKY	WRKY45	0,56	0,20	1,40	0,82	4,66	1,63
At5g46350	WRKY	WRKY8	0,38	0,84	0,94	0,59	3,82	1,16
At1g62300	WRKY	WRKY6	0,10	0,45	1,03	1,06	3,51	0,95
At4g01720	WRKY	WRKY47	0,06	0,10	0,74	0,66	2,77	0,76
At4g18170	WRKY	WRKY28	0,25	0,72	0,65	1,00	2,68	0,45
At2g38470	WRKY	WRKY33	-0,18	0,27	0,39	0,61	2,46	0,62
At5g13080	WRKY	WRKY75	0,34	0,30	3,55	1,20	8,31	1,12
At5g24110	WRKY	WRKY30	1,45	0,87	2,43	0,79	7,03	0,72
At1g29860	WRKY	WRKY71	1,35	0,71	2,02	0,92	5,91	2,17
At1g18860	WRKY	WRKY61	2,00	0,09	2,90	0,59	5,46	2,48
At1g66560	WRKY	WRKY64	0,49	1,24	1,22	0,80	5,46	1,01
At5g01900	WRKY	WRKY62	1,37	0,32	0,06	1,40	4,93	1,64
At5g22570	WRKY	WRKY38	0,67	0,30	1,40	0,52	4,92	1,54
At5g64810	WRKY	WRKY51	0,22	0,50	-0,07	1,37	4,74	1,77
At4g23810	WRKY	WRKY53	0,40	0,20	0,32	0,76	4,54	0,97
At2g40740	WRKY	WRKY55	1,22	0,59	0,07	0,81	4,13	1,54
At1g80840	WRKY	WRKY40	0,45	1,04	1,09	0,91	3,66	0,85
At1g69600	ZF-HD	ATHB29/ZFHD1/ZHD11	0,58	0,82	-0,18	0,93	4,12	1,76

**downregulated in *ap2c1 mkp1***								
			ap2c1		***mkp1***		***ap2c1 mkp1***	
**AGI**	**family**	**gene name(s)**	**-ΔΔct**	**SD**	**-ΔΔct**	**SD**	**-ΔΔct**	**SD**
At3g26790	ABI3/VP1	FUS3/FUSCA3	-0,49	0,06	-5,42	1,71	-6,84	2,39
At3g50510	AS2 (LOB) I	LBD28	-0,09	1,26	-8,09	0,51	-2,15	0,34
At4g32280	Aux/IAA	IAA29	0,60	1,17	-0,88	0,89	-3,57	0,47
At1g52830	Aux/IAA	IAA6	0,11	0,46	-1,46	0,36	-2,89	0,58
At1g04240	Aux/IAA	IAA3/SHY2	-0,16	0,58	-0,83	0,48	-2,48	0,65
At2g01200	Aux/IAA	IAA32	-0,04	0,48	-0,40	0,91	-1,83	0,67
At3g21330	bHLH		0,20	0,43	-1,32	1,22	-4,06	0,83
At1g02340	bHLH	FBI1/HFR1/REP1/RSF1	0,75	0,82	0,18	1,00	-3,31	1,17
At5g51790	bHLH		0,46	0,41	0,14	0,25	-2,69	1,13
At5g39860	bHLH	BHLH136/BNQ1/PRE1	0,56	0,82	-0,22	0,64	-2,06	0,15
At5g15160	bHLH	BHLH134/BNQ2	-0,09	0,19	-0,61	0,41	-2,03	0,68
At5g44260	C3H	TZF5	0,67	0,94	-0,71	0,98	-2,93	0,69
At3g13840	GRAS		-0,96	0,19	-0,12	0,50	-2,12	0,72
At5g27810	MADS		-0,07	1,11	-2,10	0,56	-3,32	1,03
At3g15320	MYB		-0,35	0,50	-2,39	0,75	-3,89	1,03
At5g56840	MYB-related		0,01	0,73	-0,53	1,35	-3,61	1,77
At1g75250	MYB-related	RL6/RSM3	-0,31	0,53	-1,09	0,20	-2,47	0,81
At5g43290	WRKY	WRKY49	0,97	0,00	-1,32	0,40	-3,89	1,68

Supplementary Table S5. The Arabidopsis Genome Initiative (AGI) codes of the genes analysed in this report.

At1g01010	At1g02220	At1g02250	At1g02340	At1g04240	At1g04370	At1g07160
At1g08320	At1g13300	At1g18570	At1g18860	At1g18860	At1g19210	At1g29860
At1g36060	At1g52830	At1g62300	At1g66560	At1g66560	At1g67100	At1g69600
At1g71520	At1g71860	At1g74080	At1g75250	At1g80840	At2g01200	At2g30020
At2g33710	At2g37430	At2g38250	At2g38340	At2g38470	At2g40180	At2g40470
At2g40740	At2g40740	At2g43000	At2g43140	At2g43790	At2g47520	At3g01970
At3g04070	At3g13840	At3g15320	At3g15500	At3g21330	At3g23230	At3g23240
At3g23250	At3g26790	At3g26830	At3g44350	At3g45640	At3g46080	At3g46090
At3g50510	At3g53600	At3g55270	At4g01370	At4g01720	At4g01720	At4g18170
At4g18870	At4g23810	At4g32280	At4g36990	At5g01900	At5g04390	At5g13080
At5g13330	At5g15160	At5g19520	At5g20240	At5g22570	At5g23000	At5g24110
At5g27810	At5g39860	At5g43290	At5g43650	At5g44260	At5g45890	At5g46350
At5g51790	At5g56840	At5g56960	At5g59820	At5g64750	At5g64810	At5g67450
